# Prediction of Recurrent Emergency Department Visits in Patients With Mental Disorders

**DOI:** 10.3389/fpsyt.2020.00048

**Published:** 2020-02-25

**Authors:** Ksenija Slankamenac, Raphael Heidelberger, Dagmar I. Keller

**Affiliations:** Emergency Department, University Hospital Zurich, Zurich, Switzerland

**Keywords:** recurrent emergency department users, mental disorders, personality disorders, risk factors, prediction

## Abstract

**Background:**

Patients with mental disorders are more likely to be frequent emergency department (ED) users than patients with somatic illnesses. There is little information about recurrent ED visitors (≥four ED visits/year) due to mental health problems in Switzerland. Therefore, our aim was to investigate the prevalence of recurrent ED visits due to mental disorders and to determine which mental disorders and risk factors were associated with recurrent ED visits.

**Methods:**

In a retrospective analysis, we investigated patients suffering from mental health problems between January and December 2015 who presented more than once in the ED of a tertiary care hospital. ED patients who sought out the ED due to mental disorders were grouped in a recurrent group with at least four ED visits per year or in a control group visiting the ED twice or three times within a year. The primary endpoint was to assess the prevalence of recurrent ED patients due to acute symptoms of mental disorders. As secondary endpoints, we investigated which mental disorders and risk factors were associated with recurrent ED visits.

**Result:**

Of 33,335 primary ED visits, 642 ED visits (1.9%) were by 177 visitors suffering from acute mental health problems. Forty-five (25.4%) of these 177 patients were recurrent ED visitors; 132 (74.6%) visited the ED twice or three times (control). Patients with personality disorders had a four-times higher risk (p = 0.011) of being a recurrent ED visitor. Recurrent ED visitors with mental disorders had significantly more in-house admissions (p < 0.001), self-mutilations (p < 0.001), acute drug toxicity (p = 0.007) and were more often persons of single status (p = 0.045). Although recurrent ED visitors more often had an outpatient general physician or psychiatrist, they visited the ED more frequently within office hours (p < 0.001).

**Conclusion:**

A quarter of frequent ED users with mental disorders are recurrent ED visitors and were more likely to suffer from personality disorders. Recurrent ED visits are associated with higher rates of self-mutilation, acute drug toxicity, and a greater number of in-house admissions.

## Introduction

Emergency department (ED) overcrowding is an increasing public health issue and is particularly associated with rising health care costs, longer waiting times, longer overall length of ED stay, decreasing patient satisfaction, and higher mortality (1–4). ED overcrowding has a significantly financial impact on the health care system (5, 6). Reducing ED overcrowding by increasing the transfer to inpatient clinics has potentially saved 10 million dollars in charges in US county hospitals and another 4 million dollars in costs per year in US university hospitals (5).

During the last decade, the overall number of ED visits has continuously increased worldwide, and almost two-thirds of ED users visited the ED more than once within the past year (7). These so-called frequent ED visitors account for a small number of ED patients (4.5 to 8%) but comprise 21 to 28% of all ED visits (7–9).

There is a wide range of definitions for what are termed “frequent visitors.” Most often such persons were defined as having four or more ED visits during the past 12 months, but some research groups classify patients as “frequent users” who have just two visits/year, while others set the criterion at 12 visits/year (7, 8, 10–12). Furthermore, there is also no consensus about the designation used for such a category of ED users. Some publications utilize “frequent” while others employ terminology such as “recurrent” or “repeated” visitors (7, 8, 11, 13–15). Despite these terminological differences, all articles investigated patient populations that visited the ED several times in the past year. Recently, our research group made the differentiation between “frequent” and “repeated” ED visitors due to the fact that they found significant differences in the characteristics and risk profiles within this ED population (9). “Repeated” ED users were defined as those who made a visit at least four times in 12 months for identical symptoms and health care problems, whereas “frequent” ED patients visited the ED for various diverse symptoms and issues within a 1-year period (9).

Patients visiting the ED recurrently are more likely to be suffering from chronic somatic diseases, drug and alcohol abuse, as well as mental disorders (2, 8, 9, 11, 12, 14, 16–19). Focusing on the literature about recurrent ED users with acute mental health problems, a 25–30% prevalence of acute mental illness among frequent ED users has been noted (16, 17, 20–25). Personality disorders, depression, and anxiety were the leading psychiatric diagnoses among recurrent ED users with mental disorders (16, 17, 23, 24). Reported predictors for recurrent ED visits due to acute mental health problems were chronic substance abuse, single status, homelessness, and not having a social health insurance (9, 24–26). In Switzerland, no study has been conducted investigating the subpopulation of recurrent ED users due to acute mental health problems.

Therefore, the purpose of this study was to assess the prevalence of ED visits due to mental disorders among frequent ED users and to determine which mental disorders and risk factors were associated with recurrent ED visits.

## Methods

In this retrospective study, all patients were included who visited the ED of a tertiary care hospital due to acute symptoms of mental health problems more than once between January 1^st^ and December 31^st^, 2015.

The tertiary care ED treats nearly 45,000 adult patients suffering from various disorders (e.g., internal medical problems, surgical, (poly-)trauma, head injuries, and/or psychiatric problems), and provides a full interdisciplinary and inter-professional emergency service 24/7. Of especial importance, a psychiatrist is available 24/7 in the ED; this constitutes a significant difference compared to the surrounding hospitals that do not have a psychiatric physician on staff duty in the ED. In case of acute mental health problems, every patient is treated by an interdisciplinary team of ED physicians, a psychiatrist, and ED nurses. All patients are assessed by means of a structured clinical interview and the final diagnosis of the mental disorder are made by the psychiatrist.

In this study, patients were excluded if they were younger than age 18, or visited the ED only once due to symptoms of mental health problems or for planned follow-up checks. Furthermore, patients were excluded if they suffered from mental disorders as co-morbidities but made a visit due to acute symptoms of somatic disorders. The study was approved by the local ethics committee (BASEC N° Req-2016-00195).

### Group Definitions

There are several different thresholds for “recurrent” ED visits (20, 27–31). The most common and established threshold for the definition of recurrent ED visits are four or more visits per year (8).

Therefore, patients making a visit to the ED at least four times per year with any kind of acute mental health symptoms were grouped in the *recurrent group*, whereas ED patients visiting the ED twice or three times within a the span of a year due to mental problems were allocated to the control group.

### Endpoints

The primary endpoint was to assess the prevalence of recurrent ED patients with acute symptoms of mental disorders within a subgroup of frequent ED patients of mental illness. As secondary endpoints, we investigated which mental disorders and risk factors were associated with recurrent ED visits. Additionally, we analyzed whether patients of the recurrent group had more hospital admissions due to acute symptoms of mental disorders as compared with the control group.

### Assessment and Reporting of Other Parameters

Some clinical and demographic data were recorded from the hospital digital clinical information system in order to characterize the enrolled population. The following parameters were reported: age, gender, primary diagnosis of mental disorders, co-morbidities, level of triage upon admission by the emergency severity index (ESI) (32), regular medication, regular drug abuse, domestic violence in the past, suicide attempts in the past, ED self-admittance or by paramedics or external doctors, need for fixation due to aggression to self and/or others, and symptoms leading to ED visits. Furthermore, we assessed the presence of a general physician (GP) and psychiatric specialist, and social data such as homelessness, residency (home alone, home with others), marital status (single/married/in partnership/divorced) and widow/-er, level of education (academic, professional qualification, in training, no profession) as well as professional status (differentiating between being employed, unemployed, in training, retired or disabled). Moreover, outcome parameters such as a need for hospital stay, and need for admission to a psychiatric institution were reported and analyzed.

### Statistical Analysis

We analyzed the distribution of variables using means and standard deviation (SD) for normally distributed data, and medians and interquartile ranges (IQR) for skewed data. We tested the dataset for normality employing the Kolmogorow-Smirnow test. Categorical data were presented as frequency.

The regression model was used to analyze the association between the dependent variable (outcome) and more independent variables by estimating probabilities. For this, the primary (number of recurrent ED visits) and all other secondary endpoints were compared between the recurrent ED patients (≥4 visits per year) and the control group (two or three visits within a 12-month period) using univariate and multivariable linear as well as logistic regression models. The multivariable model was adjusted for *a priori* defined and known potential confounders such as age, gender, regular drug abuse, and known domestic violence in the past. To investigate which mental disorders and potential risk factors were salient for being a recurrent ED user within this population of ED patients with mental disorders, a multivariable logistic regression analysis was performed. Furthermore, to analyze the association between ED patients having a GP or not and those having an outpatient psychiatric specialist or not, a multivariable logistic regression analysis was also used.

For all results, point estimates, 95% confidence intervals and p-values (<0.05 considered significant) were reported. The statistical analyses were performed using the statistical program STATA SE (version 15, Stata Corp., College Station, Texas).

## Results

Among 33,335 primary ED visits annually in 2015, 642 ED visits (1.9%) were made by 177 visitors suffering from mental disorders. These 177 patients visited the ED at least twice or more often due to acute symptoms of mental disorders, with a median number of two ED visits per patient (IQR 2–4). Forty-five patients (25.4%) visited the ED four times or more (recurrent group) and made 302 ED visits, constituting nearly half (47.0%) of all ED visits. An ED visitor from the recurrent group visited the ED on the median five times per year (IQR 4–8). Whereas the remaining 132 ED patients (74.6%) went to the ED twice or three times (control group) during the 1-year period and completed 340 ED visits (52.9%) due to acute symptoms of mental disorders.

### Patients’ Characteristics and General Social Factors

**Table 1** presents patients' characteristics. Patient mean age was 41 years (SD 14) old and 25.4% were female. Almost a third of all patients had been in regular outpatient therapy for underlying mental disorders. In general, patients suffered on the median from two different mental illnesses (IQR 1–3). Every third patient in the recurrent group experienced a suicide attempt in the past, whereas by contrast 18.2% of the control group had attempted suicide once. Among 15.3% of patients, an incident of domestic violence in the patient's history was reported. Additionally, 27.1% of the entire patient population was regularly on drugs.

**Table 1:** Patients’ characteristics.

	All patients N = 177	ED patients with two or three visits/year (control) N = 132	ED patients with ≥ 4 visits/year (recurrent) N = 45
Age, years	41 (14)	40.3 (13.7)	42.7 (16.0)
Sex (female) (%)	45 (25.4%)	28 (21.2%)	17 (37.8%)
Total number of underlying mental disorders (%) -Regular outpatient therapy for mental disorders in the history	2 (1–3) 58 (32.8%)	2 (1–3) 42 (31.8%)	3 (1–4) 16 (35.6%)
Suicide attempt in the past (%)	39 (22.0%)	24 (18.2%)	15 (33.3%)
Domestic violence in the history (%)	27 (15.3%)	19 (14.4%)	8 (17.8%)
Coronary heart disease (%)	7 (4.0%)	4 (3.0%)	3 (6.7%)
Diabetes mellitus (%)	11 (6.2%)	4 (3.0%)	7 (15.6%)
Arterial hypertension (%)	18 (10.2%)	8 (6.1%)	10 (22.2%)
Chronic obstructive pulmonary disease (%)	7 (4.0%)	5 (3.9%)	2 (4.4%)
Chronic kidney failure (%)	4 (2.3%)	2 (1.5%)	2 (4.4%)
Chronic liver insufficiency (%)	6 (3.4%)	6 (4.5%)	0%
HIV infection (%)	8 (4.5%)	7 (5.3%)	1 (2.2%)
Regular drug abuse (%) -Past drug abuse (%)	48 (27.1%) 22 (12.4%)	38 (28.8%) 16 (12.1%)	10 (22.2%) 6 (13.3%)
General physician available (%)	95 (53.7%)	65 (49.2%)	30 (66.7%)
Homelessness (%)	28 (15.8%)	21 (15.9%)	7 (15.6%)
Decaying condition (%)	29 (16.4%)	20 (15.2%)	9 (20%)
Housing (%) -Living alone -Living with others -Supervised living	82 (46.3%) 76 (42.9%) 19 (10.7%)	61 (46.2%) 58 (43.9%) 13 (9.8%)	21 (46.7%) 18 (40%) 6 (13.3%)
Marital status (%) -Single -Married/in partnership -Divorced -Widowed	103 (58.2%) 45 (25.4%) 27 (15.3%) 2 (1.1%)	72 (54.5%) 37 (28.0%) 22 (16.7%) 1 (0.8%)	31 (68.9%) 8 (17.8%) 5 (11.1%) 1 (2.2%)
Children (%) -Of minor age	58 (32.8%) 32 (18.1%)	44 (33.3%) 26 (19.7%)	14 (31.1%) 6 (13.3%)
Professional/vocational status (%) -Employed -Unemployed -In training -Retired -Disabled	47 (26.6%) 68 (38.4%) 7 (4.0%) 11 (6.2%) 44 (24.9%)	41 (31.1%) 46 (34.8%) 6 (4.5%) 6 (4.5%) 33 (25%)	6 (13.3%) 22 (48.9%) 1 (2.2%) 5 (11.1%) 11 (24.4%)
Level of education (%) -Academic -Professional qualification -In training -No profession -Unknown	21 (11.9%) 57 (32.2%) 7 (4.0%) 40 (22.6%) 52 (29.4%)	16 (12.1%) 41 (31.1%) 6 (4.5%) 26 (19.7%) 43 (32.6%)	5 (11.1%) 16 (35.6%) 1 (2.2%) 14 (31.1%) 9 (20%)

ED, emergency department. Results are presented as mean (standard deviation) or median (25^th^–75^th^ percentile).

In sum, the majority of the total patient population was healthy and only a small number of all patients evinced co-morbidities such as coronary heart diseases (4.0%), diabetes (6.2%), chronic obstructive pulmonary diseases (4.0%), or chronic kidney failure (2.3%) (**Table 1**).

Various social parameters are reported in **Table 1**. A general practitioner (GP) was more often recorded in the case of recurrent ED patients as compared to the control group.

The majority of patients lived alone or in a community. Patients of the recurrent group were more often singles, but a similar frequency of children was found compared to the control group. Almost half the population of the recurrent group was unemployed or in the case of 24.4%, individuals with a disability. Patients in the control group were mostly employed or jobless. In both groups, a third of the patients had completed a professional or vocational training course, whereas among recurrent patients 31.1% were without any profession (**Table 1**).

### Presentation in the Emergency Department

**Table 2** presents patients' parameters upon admission to the ED. Of a total 642 ED visits, 502 (78.2%) were triaged upon ED admission to ESI level three (**Table 2**). Patients of the recurrent group were triaged less often as ESI level 3 (74.2 *vs*. 81.8%) as compared to the control group (**Table 2**).

**Table 2:** Some parameters upon admission as attender/presenter to the emergency department (ED).

	All ED visits N = 642	Two or three ED visits/year N = 340	≥ 4 ED visits/year N = 302
Triage level upon ED admission by the ESI (%) -ESI 1 -ESI 2 -ESI 3 -ESI 4/5	9 (1.4%) 51 (7.9%) 502 (78.2%) 80 (12.5%)	4 (1.2%) 21 (6.2%) 278 (81.8%) 37 (10.9%)	5 (1.7%) 30 (9.9%) 224 (74.2%) 43 (14.2%)
Number of ED visits during: -8 am–5 pm -5 pm–11 pm -11 pm–8 am	1 (1–2) 1 (0–2) 1 (0–1)	1 (0–2) 1 (0–1) 0 (0–1)	3 (2–4) 2 (1–3) 1 (0–3)
Number of self-admittances	2 (1–3)	2 (1–2)	4 (1–6)
Number of admittances by paramedics	1 (0–2)	0 (0–2)	1 (0–4)
Number of emergency allocations by external doctors	0 (0–1)	0 (0–1)	0 (0–2)
ED admission with police (%)	22 (3.4%)	16 (4.7%)	6 (2.0%)
Need for fixation strategies in the ED (%)	6 (0.9%)	4 (1.1%)	2 (0.7%)

ED, emergency department; ESI, Emergency Severity Index. Results are presented as median (25^th^–75^th^ percentile).

Furthermore, the recurrent patients had more ED visits made during daytime and late shifts compared to the control group. They also came for a visit more often by self-admittance than via vocational the agency of paramedics (**Table 2**).

### Leading Symptoms and Reasons for Emergency Department Visits

**Table 3** presents leading symptoms and reasons encountered for ED visits. The three leading acute mental symptoms for frequent ED visits were aggression (20.3%), feeling down, sad or miserable (18.1%), and hallucinations (17.5%). Whereas by contrast, patients of the recurrent group visited the ED repeatedly due to different withdrawal symptoms (26.7%), hallucinations (22.2%), and aggression (17.8%). Additionally, eight recurrent ED patients (17.8%) suffered from chest pain and had negative cardiac diagnostic test results for acute myocardial infarction during examinations in the ED. In addition, no organic etiology for chest pain was found in any of the eight patients complaining of chest pain.

**Table 3:** Symptoms or reasons leading to emergency department (ED) visits.

	All patients N = 177	ED patients with two or three visits/year N = 132	ED patients with ≥ 4 visits/year N = 45
**Different symptoms**			
Aggression (%)	36 (20.3%)	28 (21.2%)	8 (17.8%)
Feeling down, sad, or miserable (%)	32 (18.1%)	28 (21.2%)	4 (8.9%)
Hallucinations (%)	31 (17.5%)	21 (15.9%)	10 (22.2%)
Anxiety or panic (%)	24 (13.6%)	19 (14.4%)	5 (11.1%)
Different withdrawal symptoms (%)	22 (12.4%)	10 (7.6%)	12 (26.7%)
Increased stress load (%)	17 (9.6%)	13 (9.8%)	4 (8.9%)
Chest pain (%)	13 (7.3%)	5 (3.8%)	8 (17.8%)
Insomnia (%)	6 (3.4%)	4 (3.0%)	2 (4.4%)
Others* (%)	69 (39.0%)	39 (29.5%)	30 (66.7%)
**Different reasons**			
Acute drug toxicity (%) -Only alcohol -Drugs and alcohol	90 (50.8%) 61 (34.5%) 29 (16.4%)	62 (47.0%) 45 (34.1%) 17 (12.9%)	28 (62.2%) 16 (35.6%) 12 (26.7%)
Suicide attempt (%)	54 (30.5%)	38 (28.8%)	16 (35.6%)
Self-mutilation (%)	45 (25.4%)	24 (18.2%)	21 (46.7%)
Foreign body invagination (%)	4 (2.3%)	1 (0.8%)	3 (6.7%)
Wish for a medical prescription (%)	14 (7.9%)	9 (6.8%)	5 (11.1%)
Wish for alcohol or drug withdrawal (%)	12 (6.8%)	6 (4.5%)	6 (13.3%)
Wish for psychiatric admission (%)	8 (4.5%)	4 (3.0%)	4 (8.9%)

It is possible that more than one symptom and/or reason leading to ED visit; ED, emergency department. Others included symptoms of generalized or specific pain (e.g., headache, back, limbs, or abdominal pain), poor appetite, weakness, fatigue, dizziness, trembling or muscle cramps, etc.

The most frequent reason for ED visits was acute drug toxicity (50.8%). Recurrent patients had far more ED visits due to a combination of drug and alcohol toxicity (26.7 *vs*. 12.9%, adjusted OR 4.2, 95% CI 1.5–12.0, p = 0.007) compared to the control group. The rate of acute drug toxicity due solely to alcohol overdose was similar in both groups (adjusted OR 0.97, 95% CI 0.5–2.0, p = 0.93).

Furthermore, recurrent ED patients did not visit significantly more often due to the consequences of a suicide attempt (35.6 *vs*. 28.8%, adjusted OR 1.6, 95% CI 0.7–3.3, p = 0.26). But they visited the ED significantly more often due to the consequences of self-mutilation (46.7 *vs*. 18.2%, adjusted OR 4.2, 95% CI 1.9–8.9, p < 0.001) as compared to patients in the control group.

Only a small number of patients visited the ED frequently with the wish to obtain a medical prescription, for alcohol or drug withdrawal, or desired psychiatric admission (**Table 3**).

### Diagnosis of Mental Disorders Leading to Recurrent Emergency Department Visits

Assigning these multiple symptoms to a diagnosis of mental disorders (**Table 4**), the majority of patients visited the ED as a result of psychoactive substance addiction (41.8%). The drug addiction was similarly distributed in both groups.

**Table 4:** Leading diagnoses of mental disorders encountered.

	ED patients with two or three visits/year N = 132	ED patients with ≥ 4 visits/year N = 45	Unadjusted OR (95% CI, p-value)	Adjusted OR (95% CI, p-value)
Psychoactive substance or medical addiction (%)	54 (40.9%)	20 (44.4%)	1.2 (0.7–2.9, p = 0.78)	1.1 (0.5–2.4, p = 0.90)
Schizophrenia, schizotypal, delusional, and other non-mood psychotic disorders (%)	22 (16.7%)	5 (11.1%)	0.6 (0.2–1.8, p = 0.37)	0.6 (0.2–1.8, p = 0.37)
Anxiety, dissociative, stress-related, and other non-psychotic mental disorders (%)	23 (17.4%)	3 (6.7%)	0.3 (0.1–1.2, p = 0.09)	–
Personality disorders (%)	12 (9.1%)	10 (22.2%)	2.9 (1.1–7.2, p = 0.025)	4.0 (1.4–11.8, p = 0.011)
Affective disorder (%)	19 (14.4%)	3 (6.7%)	0.4 (0.1–1.5, p = 0.19)	–
Somatoform disorders (%)	2 (1.5%)	4 (8.9%)	6.3 (1.1–35.9, p = 0.037)	–

ED, emergency department; OR, odds ratio; CI, confidence interval. Results are adjusted for age sex, regular drug consumption, and known domestic violence in the past. No adjustment was performed if fewer than five cases occur in one of the groups.

Furthermore, personality disorders were significantly more often represented among recurrent ED patients (adjusted OR 4.0; 95% CI 1.4–11.8, p = 0.011). A trend was also identified for somatoform disorders (unadjusted OR 6.3, 95% CI 1.1–35.9, p = 0.037), but was weakened by the presence of too few events in both groups (**Table 4**).

### Social Risk Factors Leading to Recurrent Emergency Department Visits

Analyzing social parameters as potential risk factors for recurrent ED visits due to acute mental symptoms, being single was identified as the only significant predictor (adjusted OR 2.2, 95% CI 1.1–4.8, p = 0.045).

Being unemployed and the fact of never have learned a profession/vocation evinced a trend for increased recurrent ED visits; however, this was not significant (**Table 5**).

**Table 5:** Differences and risks for recurrent emergency department (ED) visits.

	ED patients with two or three visits/year N = 132	ED patients with ≥ 4 visits/year N = 45	Unadjusted OR (95% CI, p-value)	Adjusted OR (95% CI, p-value)
Single status (%)	72 (54.5%)	31 (68.9%)	1.8 (0.9–3.8, p = 0.10)	2.2 (1.1–4.8, p = 0.045)
Unemployed (%)	46 (34.8%)	22 (48.9%)	1.7 (0.9–3.5, p = 0.10)	1.8 (0.9–3.6, p = 0.16)
No profession/vocation (%)	26 (19.7%)	14 (31.1%)	1.8 (0.9–3.9, p = 0.12)	2.0 (0.9–4.4, p = 0.09)
General physician available (%)	65 (49.2%)	30 (66.7%)	2.0 (1.01–4.2, p = 0.045)	2.1 (1.1–4.4, p = 0.043)
Regular psychiatric outpatient visits (%)	42 (31.8%)	16 (35.6%)	1.2 (0.6–2.4, p = 0.65)	1.3 (0.6–2.7, p = 0.54)
			**Unadjusted difference (95% CI, p-value)**	**Adjusted difference (95% CI, p-value)**
Number of ED visits during 8 am to 5 pm	1 (0–2)	3 (2–4)	1.5 (1.0–1.9, p < 0.001)	1.5 (1.1–1.9, p < 0.001)
Number of ED visits during 5 to 11 pm	1 (0–1)	2 (1–3)	1.3 (0.9–1.7, p < 0.001)	1.3 (0.9–1.7, p < 0.001)
Number of ED visits during 11 pm to 8 am	0 (0–1)	1 (0–3)	1.3 (0.8–1.8, p < 0.001)	1.4 (0.9–1.9, p < 0.001)

ED, emergency department; OR, odds ratio; CI, confidence interval. Results were presented as median (25th–75th percentile). Results are adjusted for age sex, regular drug consumption and known domestic violence in the past.

### Inpatient Admissions due to Acute Symptoms of Mental Disorders

Patients of the recurrent group were significantly more often admitted to an inpatient clinic (2 (IQR 0–4) *vs*. 1 (IQR 0–2); adjusted difference 1.6, 95% CI 1.1–2.0, p < 0.001) as a result of acute symptoms. They were also significantly more often admitted to psychiatric clinics (1 (IQR 0–2) *vs*. 1 (IQR 0–1; adjusted difference 0.8, 95% CI 0.4–1.2, p < 0.001).

### Prevalence of General Physician or Outpatient Psychiatrist

In total, 95 patients (53.6%) reported when admitted to the ED that they had a GP. Recurrent ED patients had a GP significantly more often than as compared to the control group (95% CI 1.1–4.4, p = 0.043).

Furthermore, 58 patients (32.8%) even had an outpatient psychiatrist. There was no difference between the two groups in the rate of psychiatrists (95% CI 0.6–2.7, p = 0.54) (**Table 5**).

Although recurrent ED visitors had more often a GP or psychiatrist, they presented more ED visits within office hours (adjusted difference 1.5, 95% CI 1.1–1.9, p < 0.001) than the control group (**Table 5**).

## Discussion

A quarter of frequent ED users with mental disorders are recurrent ED visitors and were more likely to suffer from personality disorders. Recurrent ED visits are associated with higher rates of self-mutilation, acute drug toxicity, and more in-house admissions. A social risk factor such as being single is more likely to increase the rate of recurrent ED visits. Although recurrent ED visitors more often had an outpatient general physician or psychiatrist, they visited the ED more frequently within office hours.

The general population of frequent ED visitors suffers more often from psychiatric disorders, pain complaints and more frequently, psychoactive substances were misused and the leading cause for recurrent ED visits (2, 8, 9, 11, 12, 14, 16–19). A prevalence of recurrent ED visits of 28–30% has been reported in ED patients making frequent visits due to acute symptoms of mental health problems (16, 17, 33). The prevalence of 25.4% in this current study corresponds with findings in the international literature.

Further findings of this current Swiss study are comparable with the findings in the literature (16, 17, 20–25). Personality disorders, depression and anxiety disorders were the leading psychiatric diagnoses among recurrent ED users with mental disorders (16, 17, 23, 24). Among these, personality disorders were reported to be the most frequent mental illness in recurrent ED users (11, 16, 17, 25). Vandyk et al. reported that 9 of 10 recurrent ED patients with mental disorders visited the ED due to acute symptoms or consequences of antisocial or borderline personality disorder (25). Less frequent but still a high number of borderline effects were recorded in the current study. Almost every second patient presented recurrently in the ED due to consequences of self-mutilation. We reported psychoactive substance addiction, personality disorders, and schizophrenia as the three leading diagnoses for recurrent ED visits. Finally, only ED patients with personality disorders were more likely to have recurrent ED visits. This can be explained by the long-standing course of the disease and its multiple and recurrent exacerbations that lead patients with personality disorders to recurrent ED visits (25, 34). The ED is indeed the accurate place to treat acute consequences of personality disorders by treating cuts and wounds or removing foreign bodies from any body site, for example, but the ED is not the right locus for treatment of the chronic underlying psychiatric disease. Over the long term, it is unclear how ED physicians may assist persons in this vulnerable patient population. In 2017, a randomized controlled trial was performed to investigate whether a brief case management organized by the ED can support these patients with psychiatric diagnosis in order to reduce the number of ED visits (35). Stergiopoulos et al. could not show any effect of the case management on the number of ED visits. A possible solution to reduce the large number of ED visits may be through extended collaboration with outpatient psychiatric services (33). For this specific issue, special outpatient therapies and close support by psychiatrists are needed in the future.

Minassian et al. found that recurrent ED visits due to acute mental health problems were related to alcohol abuse (26). Several articles identified that substance abuse was significantly associated with mental disorders and multiple ED presentations (20, 23, 33, 36). These results reflect the complexity of special needs of these patients and suggest that a well-trained and interdisciplinary team of ED physicians, psychiatrists, and ED nurses need to provide care for this specific population. In the current study, sole alcohol overuse was not associated with recurrent ED visits, whereas drug intake in combination with alcohol led significantly more often to recurrent ED visits. More than 60% of the recurrent patients visited the ED due to acute symptoms of psychoactive substance misuse. The rates for acute symptoms with the need for ED admission due to an alcohol or psychoactive drug overuse are similar in the literature to our current findings (17, 20, 23, 33, 36).

There is a high prevalence of substance abuse (20–25%) in the medical history of recurrent ED patients with mental symptoms (12, 17, 20, 23, 26, 33, 36). The current study also reports that every fifth recurrent ED patient had used drugs regularly in the past.

Acute symptoms of schizophrenia leading to recurrent ED visits were mostly reported among highly frequent ED users (>11 or even 18 visits/year) (17, 18). In the current sample, acute symptoms and consequences of schizophrenia were the third most frequent reason for recurrent ED visits, but there was no significant association between the recurrent ED visitors suffering from schizophrenia and an increased rate of ED visits. Whether schizophrenia is a risk factor in the highly frequent ED population with mental illness is unclear and could not be statistically analyzed in the current study. Only 13 patients had 8 or more ED visits due to acute symptoms of mental disorder. None of these 13 patients suffered from schizophrenia. Therefore, more research is needed in this rare population of highly frequent ED visitors to better understand those vulnerable populations and to identify potential supporting measures.

Recurrent ED patients were more likely to be hospitalized (19–28%) than general ED users (14–16%) (9–11, 33, 37). Hansagi et al. found that 80% of recurrent ED patients needed hospital admission (38). The authors argued that the high rate for hospital admission was due to a large number of severely ill patients with co-morbidities (38). Recurrent ED users with psychiatric disorders have in general fewer co-morbidities and are therefore considered healthier overall. Thus, this might suggest that recurrent ED users suffering from acute mental problems have a lower rate of hospital admission. This was not the case. In the literature as well as in this current study, recurrent ED users with acute symptoms of mental illness showed a significantly higher number of hospital admissions and admissions to psychiatric clinics compared to the control group. Indeed, those patients were healthier and showed a lower rate of co-morbidities, but they also suffered more frequently from acute symptoms due to alcohol or substance abuse or consequences of self-mutilation that led to a greater number of hospital admissions (33, 36, 39).

The chronic nature of mental disorders impairs the social and occupational functioning, and in addition to the increased rates of exacerbating factors, different social factors were shown to be predictors for recurrent ED presentations. Being a single parent, having single or divorced marital status, being unemployed, having a high school education level or lower and/or low income were associated with an increased number of ED presentations (9, 19, 25, 33, 36). In addition, Chang et al. pointed to other social factors such as homelessness and no social health insurance as predictors for recurrent ED use by patients with acute mental illness (24). In the current study, being of single status was the only social predictor for recurrent ED use. Furthermore, homelessness, divorced marital status, level of education, and professional/vocational status could not be identified as social predictors because the sample in each group was too small. Social health insurance was not evaluated as a predictor because in Switzerland, every patient is required to have at least a general insurance policy that covers medical expenses. Further studies with larger sample sizes and prospective recording of further social factors, such as income, indebtedness, social problems in school, the family, or workplace, are needed.

### Strengths and Limitations

There are several limitations in the study conducted. First, it is a single-center study in a region with several surrounding hospitals. Thus, it is likely that the number of recurrent ED patients is overall much higher than reported. Furthermore, recurrent ED patients are likely to pay visits to multiple EDs due to identical problems. This limitation might be negligible, because the surrounding hospitals do not have a psychiatrist in the ED available 24/7. Therefore, as a tertiary care hospital with an interdisciplinary team of ED physicians, a psychiatrist on duty, and ED nurses available on a 24/7 basis, it is the largest sample size of recurrent ED patients with mental disorders in our region, thus representing well the generalizability of our findings.

Furthermore, because of the availability of an electronic clinical information system, the study has only few missing data, and none in the endpoints.

Third, so as to reduce the bias, a randomization was not possible to conduct due to the retrospective nature of the study design. Therefore, we adjusted all our results for potential confounders by using a multivariable regression model. The strict inclusion criteria additionally reduced the selection bias in respect to recurrent ED visitors.

The focus on the subgroup of recurrent ED patients with mental disorders strengthens the findings as a result of the homogeneity of the specified population of vulnerable ED patients.

## Conclusion

Recurrent ED patients suffering from acute symptoms of mental disorders are a rare subgroup of ED patients but they constitute a fourth of the recurrent ED visitors. Recurrent ED visitors were more likely to suffer from personality disorders. Recurrent ED visits are associated with higher rates of self-mutilation, acute drug toxicity, and more in-house admissions. A possible solution to reduce the ED frequency may be a case management approach for recurrent ED patients with mental disorders. Further prospective studies are required to optimize the future patient-centered care of recurrent ED visitors.

## Author’s Note

The abstract with preliminary results of the current study was presented as a poster at: The Annual Conference of Swiss Psychiatrists (“PSY Congress”), Berne, Switzerland, September 5–7, 2018; the 12th European Congress of Emergency Medicine (EUSEM), Glasgow, United Kingdom, September 8–12, 2018; the 13th Annual Conference, Deutsche Gesel lschaft Interdisziplinäre Notfall- und Akutmedizin (DGINA), Leipzig, Germany, September 27–29, 2018; abstract published in Notfall Rettungsmed 2018, 21: 1–22. Preliminary results were presented orally at the Annual Conference, Deutsche Gesellschaft für Psychiatrie und Psychotherapie, Psychosomatik und Nervenheilkunde (DGPPN), Berlin, Germany, November 28 to December 1, 2018, oral presentation.

## Data Availability Statement

The datasets for this article are not publicly available because the ethic committee decision is only positive for the publication of the results and not for the public availability of the dataset.

## Ethics Statement

The studies involving human participants were reviewed and approved by the Ethics Committee of the Canton Zurich, Switzerland. Written informed consent for participation was not required for this study in accordance with the national legislation and the institutional requirements.

## Author Contributions

Conceptualization: KS, DK. Data curation: KS, RH, DK. Formal analysis: KS. Funding acquisition: KS. Investigation: KS, DK. Methodology: KS, DK. Project administration: KS, DK. Supervision: KS, DK. Visualization: KS, RH, DG. Writing – original draft: KS, DK. Writing – review and editing: KS, RH, DK.

## Funding

Promedica Foundation, Chur and Career grant by the University Hospital Zurich, Switzerland to KS.

## Conflict of Interest

The authors declare that the research was conducted in the absence of any commercial or financial relationships that could be construed as a potential conflict of interest.
